# Long-Term Effects of TCM Yangqing Kangxian Formula on Bleomycin-Induced Pulmonary Fibrosis in Rats via Regulating Nuclear Factor-*κ*B Signaling

**DOI:** 10.1155/2017/2089027

**Published:** 2017-12-07

**Authors:** Meng Li, Ya Li, JianSheng Li, Peng Zhao, Yunping Bai, SuXiang Feng, Xuefang Liu, Yang Wang, Qingqing Bian, Junzi Li

**Affiliations:** ^1^Dongzhimen Hospital, Beijing University of Chinese Medicine, Beijing 100700, China; ^2^Collaborative Innovation Center for Respiratory Disease Diagnosis and Treatment & Chinese Medicine Development of Henan Province, Henan University of Chinese Medicine, Zhengzhou, Henan 450000, China; ^3^Institute for Respiratory Diseases, The First Affiliated Hospital, Henan University of Chinese Medicine, Zhengzhou, Henan 450000, China; ^4^Central Laboratory, The First Affiliated Hospital, Henan University of Chinese Medicine, Zhengzhou, Henan 450000, China; ^5^Institute for Geriatrics, Henan University of Chinese Medicine, Zhengzhou, Henan 450000, China

## Abstract

**Objective:**

We aimed to evaluate the therapeutic effects and long-term effects of YKF and dissect the potential mechanisms.

**Materials and Methods:**

IPF rats were given YKF, prednisone, or pirfenidone, respectively, from day 1 to day 42, followed by a 28-day nonintervention interval through day 70. Forced vital capacity (FVC), histopathology, hydroxyproline (HYP) contents, lung coefficient, blood inflammatory cell populations, inflammatory cytokine levels of the lung tissues, and the expression of proteins involved in nuclear factor- (NF-) *κ*B signaling pathway were evaluated on days 7, 14, 28, 42, and 70.

**Results:**

HYP contents, Ashcroft scores, lung coefficient, and pulmonary fibrosis blood cell populations increased significantly in IPF rats, while FVC declined. All the above-mentioned parameters were improved in treatment groups from day 7 up to day 70, especially in YKF group. The mRNA and protein expressions of tumor necrosis factor- (TNF-) *α* significantly decreased, while interferon- (IFN-) *γ* significantly increased, and phosphorylations of cytoplasm inhibitor of nuclear factor kappa-B kinase *β* (IKK*β*), inhibitor of nuclear factor kappa-B *α* (I*κ*B*α*), and NF-*κ*B were obviously downregulated in YKF group from day 7 to day 70.

**Conclusion:**

YKF has beneficial protective and long-term effects on pulmonary fibrosis by anti-inflammatory response and alleviating fibrosis.

## 1. Introduction

Idiopathic pulmonary fibrosis (IPF) is an insidious and progressive disorder characterized by the aberrant deposition of extracellular matrix, which leads to irreversible destruction of lung architecture and dysfunction of gas exchange [1]. Recent studies suggested an increasing prevalence and increasing incidence of IPF [2, 3]. It is a fatal disease with a median survival estimated at 2–5 years from diagnosis. Nevertheless, currently available treatments, represented by pirfenidone, proved to have side effects and brought weighty economy burden [4, 5]. Therefore, novel therapeutic agents are needed urgently for the effective treatment of IPF.

IPF is a severe result of an aberrant injury repair process in lung tissue. This pathophysiologic dysregulation involves a complex interaction between epithelial injury, oxidative stress, coagulation disturbances, and inflammation, ultimately leading to transformation of several cell types into myofibroblasts and extracellular matrix deposition [6]. When encountering the invaders, alveolar epithelium can secrete tumor necrosis factor- (TNF-) *α*, the mediator of inflammatory signal pathway [7], which can amplify the inflammation response by the activation of nuclear factor- (NF-) *κ*B transcription factor. The inflammation response is responsible for the recruitments of leukocyte and lymphocyte and the aggravation of oxidative stress injury [8]. Data demonstrate that TNF-*α* is elevated in bronchoalveolar aspirate of patients with IPF [9]. Interferon- (IFN-) *γ* is an important Th-1 cytokine, which inhibits fibroblast proliferation and collagen accumulation in in vitro and in vivo studies [10, 11].

Traditional Chinese medicine (TCM) has provided effective therapies of chronic pulmonary disorders for thousands of years, including alleviating the clinical symptoms, improving pulmonary function, and exercise capacity [12, 13]. Long-term therapeutic superiority is the salient feature of TCM treatment. Current studies of traditional medicinal formula and herbal monomer were proved to be effective in the treatment of IPF [14–19]. For instance, Buzhong Yiqi formula can suppress inflammation through regulating immune response, Yu Ping Feng formula can alleviate pulmonary fibrosis by reducing the expression of transforming growth factor- (TGF-) *β*1, *α*-smooth muscle actin (SMA), and collagen (COL)-1. In addition, our previous studies have confirmed that TCM formulae display long-term beneficial effect on chronic pulmonary disease probably by modulating the lipid metabolism, oxidative stress, and inflammatory response pathways at system level [20–24]. In clinical treatment of IPF patients, we found that Yangqing Kangxian formula (YKF) had beneficial effects on alleviating the clinical symptoms of IPF patients, whose HRCT featured as interstitial pneumonia. However, the long-term effects and underlying therapeutic mechanism of YKF on IPF are not clear.

In this study, we observed the forced vital capacity (FVC), pulmonary histomorphology, hydroxyproline (HYP) contents, inflammatory biomarkers in blood and lung tissues, and activation of NF-*κ*B signaling pathway and aimed to investigate the mechanisms of YKF therapeutic effect.

## 2. Materials and Methods

### 2.1. Drugs

Yangqing Kangxian formula, a formula of traditional Chinese herbs, was prepared by the Pharmaceutical Department in Henan University of Chinese Medicine. The main compositions of YKF were shown in Table 1. All herbs were water- or ethanol-extracted and made into dry extract, ultimately, according to its standard operation procedure. Each 1 g dry extract contains 3.01 g of raw herbs.

Bleomycin was purchased from Nippon Kayaku (Batch number 730342). Prednisone acetate tablets were purchased from Zhejiang Xianju Pharmaceutical Co., Ltd. (Batch number 140138). Pirfenidone was donated by Beijing Continent Pharmaceutical Co., Ltd. (Batch number 150603).

### 2.2. Animals

Two hundred Sprague Dawley rats (weighting 180 ± 20 g, Certificated: SCXK (Yu) 2010-0002) purchased from Laboratory Animal Center of Henan Province (Zhengzhou, Henan, China) were housed in individual ventilated cages (Fengshi, Jiangsu, China) located in the First Affiliated Hospital, Henan University of Chinese Medicine, with free access to purified water and pellet feed (Xietong Medical Bioengineering, Nanjing, Jiangsu, China). The laboratory temperature was maintained at 22~24 degrees Celsius (°C), and relative humidity at 50%~70%. All rats were adapted and housed in the laboratory 5 days before experiment. The experimental procedures were approved by the Experimental Animal Care and Ethics Committees of the First Affiliated Hospital, Henan University of Traditional Chinese Medicine (Zhengzhou, Henan, China).

### 2.3. Model Preparation and Administrations

All rats were randomly divided into control group (*n* = 40), BLM group (*n* = 40), BLM + YKF group (*n* = 40), BLM + PD group (*n* = 40), and BLM + PF group (*n* = 40). The control rats were intratracheally injected with phosphate-buffered saline (PBS) via intubation after being anesthetized with 10% chloral hydrate. The other rats were intratracheally injected with bleomycin (5 mg/kg) dissolved in PBS [25]. The control and BLM rats were treated by normal saline, and the other three groups were given YKF (0.89 g/100 g body weight), prednisone (0.5 mg/100 g body weight), and pirfenidone (5 mg/100 g body weight), respectively [15, 26]. All the treatments were initiated 24 hours (day 1) after bleomycin challenge, q.d., for 42 days, and then administrations ceased from day 43 through 70. The experiment protocol was shown in Table 2. Dose adjustments were made weekly according to the body mass. The dosage of YKF was calculated according to the body surface area conversion equation: *D*_rat_ = *D*_human_ × (*I*_rat_/*I*_human_) × (*W*_rat_/*W*_human_)^2/3^: *D*: dose; *I*: body shape index; *W*: bodyweight.

Six rats were sacrificed in each group on days 7, 14, 28, and 42, the rest were sacrificed on day 70, and the samples were harvested to prepare for analysis.

### 2.4. FVC Test

FVC was determined with a computer controlled pulmonary function test (PFT) system (BUXCO, DSI, MN, USA). After being anaesthetized and endotracheally intubated, rats were placed in the sealed chamber and connected to the device via the intubation, and the respiratory data was acquired with a pressure volume transducer and presented with FlexiVent software (BUXCO, DSI, MN, USA).

### 2.5. Blood Cytological Analysis

After FVC test, the blood samples were collected from the anesthetized animals. The numbers of blood inflammatory cells were analyzed by automated differential cell counter (Beckman Coulter A^c^.T™ 5 diff, US) in our lab.

### 2.6. Lung Coefficient Calculation

After being removed and cleaned with ice-cold PBS solution, all lung lobes were wiped with filter paper and lung wet weight was weighed. Lung coefficient was calculated as the ratio of lung wet weight (mg) and body weight (g).

### 2.7. Hydroxyproline Assay in Lung Tissue

HYP contents were measured according to the manufacture's instruction of the kit (Jiancheng, Nanjing, China). 80 mg lung tissues were hydrolyzed with 1 ml of alkaline hydrolysate and boiled at 95°C for 20 min and then centrifuged at 3000 rpm for 10 min at 4°C. The supernatant was obtained and hydroxyproline content was measured on an ultraviolet spectrophotometer (Thermo Fisher, MA, US). Results were expressed in microgram per gram tissue (*μ*g/g tissue).

### 2.8. Histomorphology and Immunohistochemical Analyses

The trachea was cannulated, and the lung was removed from the thoracic cavity. The right extrapulmonary bronchus was ligated with sutures, and the right lung lobes were removed. The left lung lobe was perfusion-fixed with 10% neutral buffered formalin via the trachea at a constant pressure of 30 cm fixative for 2 h, and it was immersed in the same fixative for 72 h before further processing. After formalin fixation, the left lung lobe was cut into 3 mm thick tissue block and embedded in paraffin (Leica, Germany) after graded ethanol dehydration and xylene hyalinization. Five *μ*m thick sections were sliced and stained with standard hematoxylin and eosin (HE) solution (Solarbio, Beijing, China), and Masson's Trichrome stain kit (Solarbio, Beijing, China) according to the instructions. Histomorphological changes were inspected under a microscope (Leica, Germany), and three nonoverlapping microphotographs were captured per lung for image analysis by two researchers in a blinded fashion. Ashcroft score was assessed to evaluate the degree of pulmonary fibrosis, as follows: 0: normal lung; 1: minimal fibrous thickening of alveolar or bronchiolar walls; 3: moderate thickening of walls without obvious damage to lung architecture; 5: increased fibrosis with definite damage to lung structure and formation of fibrous bands or small fibrous masses; 7: severe distortion of structure and large fibrous areas (“honeycomb lung” is placed in this category); 8: total fibrous obliteration of the field [27].

For immunohistochemical analysis, sections were blocked with 5% bovine serum albumin (BSA) for 20 min and incubated with antibodies against TNF-*α* (1 : 150 dilution, Bioss, Beijing, China) and IFN-*γ* (1 : 100 dilution, Bioss, Beijing, China) at 4°C for 12 h, followed by incubation with goat anti-rabbit immunoglobulin G (ZSGB-BIO, Beijing, China) at 25°C for 2 h; then the sections were counterstained with hematoxylin. The expressions of the above-mentioned proteins were observed with a Leica microscope, and images were collected for semiquantitative analysis achieved by Image-Pro Plus 6.0 professional image acquisition and analysis system (Media Cybernetics, MD, USA). Three nonoverlapping microphotographs were captured per lung for image analysis by two researchers in a blinded fashion. The IHS score was calculated as Robert's report [28]: (1) Positive cell quantity includes the following: no staining scored as 0, 1~10% of cells stained scored as 1, 11~50% as 2, 51~80% as 3, and 81~100% as 4. (2) Staining intensity was rated with a scale of 0 to 3: 0 = negative; 1 = weak; 2 = moderate, and 3 = strong. When there is multifocal immunoreactivity and there are significant differences in staining intensities between foci, the average of the least intense and most intense staining was recorded. The raw data were converted to IHS by multiplying the quantity and staining intensity scores.

### 2.9. Real-Time Polymerase Chain Reaction Analysis

The expressions of TNF-*α* and IFN-*γ* mRNAs of lung tissues were analyzed by quantitative real-time polymerase chain reaction (qRT-PCR). Total RNA was extracted by using TRIzol reagent (Ambion, California, US) according to the instructions; concentration and integrity of total RNA were verified by a NanoDrop2000 nanospectrophotometer (Thermo, MA, USA) and electrophoresis in 2% agarose gel. Reverse transcription (RT) was proceeded by using SuperScript® III First-Strand Synthesis SuperMix for qRT-PCR Kit (Invitrogen, California, US), and real-time PCR reactions were performed by using Platinum® SYBR® Green qPCR SuperMix-UDG with ROX Kit (Invitrogen, California, US). The reaction systems were prepared following the instructions of the kits and reacted on an ABI 7500 real-time instrument (ABI, California, US). The initial enzyme activation step was at 95°C for 2 min, followed by 40 cycles of 95°C for 15 s and 60°C for 30 s. At the end of PCR, to evaluate specific amplification of the target genes, melting curves ranging from 60 to 95°C were also included in each run. The primers of TNF-*α* and IFN-*γ* were designed and synthesized by Genscript Biotech Co. Ltd (Nanjing, Jiangsu, China). Sequences were shown in Table 3.

### 2.10. Western Blotting Analysis

Lung tissues homogenized in radioimmunoprecipitation assay (RIPA) lysis buffer containing phenylmethanesulfonyl fluoride (PMSF) and phosphatase inhibitors (Solarbio, Beijing, China) were centrifuged at 12000 rpm for 5 min at 4°C, for total and nuclear protein extraction. The phosphorylations of I*κ*B*α* (p-I*κ*B*α*) and IKK*β* (p-IKK*β*) and nuclear phosphorylated NF-*κ*B (p-NF-*κ*B) in the lungs were determined with Western blotting technology. The protein concentrations were detected according to the instruction of BCA protein assay kit (Solarbio, Beijing, China), and then protein denaturalization was performed at 100°C for 10 min with 2% SDS and 5% 2-mercaptoethanol added. 30 *μ*g protein was separated by 10% sodium dodecyl sulfate-polyacrylamide gel electrophoresis (SDS-PAGE) and transferred to polyvinylidene difluoride (PVDF) membranes (Millipore, Bedford, MA, USA). The membranes were blocked with 5% skim milk and then incubated with primary antibody, p-NF-*κ*B, p-I*κ*B*α*, p-IKK*β* (CST, MA, USA), GADPH (Proteintech, Wuhan, China), and horseradish peroxidase- (HRP-) conjugated secondary antibodies (Proteintech, Wuhan, China) subsequently. Signals were gained by using the Super ECL Plus reagent (Solarbio, Beijing, China) and were scanned and quantified by Molecular Imager® Gel Doc™ XR System (Bio-Rad, CA, US).

### 2.11. Statistical Analysis

Unless noted, six rats were included in statistical analysis in each group on days 7, 14, 28, and 42. On day 70, four rats in BLM group, five in BLM + PD group, and six rats in the other three groups, respectively, were included in statistical analysis.

Statistical data are expressed as mean ± standard error of mean (SEM). Kruskal-Wallis *H* test was used if the data do not comply with the normal distribution; if the data comply with the normal distribution, one-way analysis of variance followed by SNK or Dunnett's T3 post hoc test was used for multiple comparisons. Differences were considered to be significant at *P* values < 0.05. All statistical analyses were performed with SPSS 19.0 software (IBM, NY, USA).

## 3. Results

### 3.1. Mortality

From day 0 to day 14, the rats of BLM group and treatment groups died of severe pneumonia induced by bleomycin. From day 15 to day 28, two rats died of choke when given gavage in BLM + YKF and BLM + PF group (Table 4).

### 3.2. YKF Ameliorated Pulmonary Function in Rats

**Figure 1:**
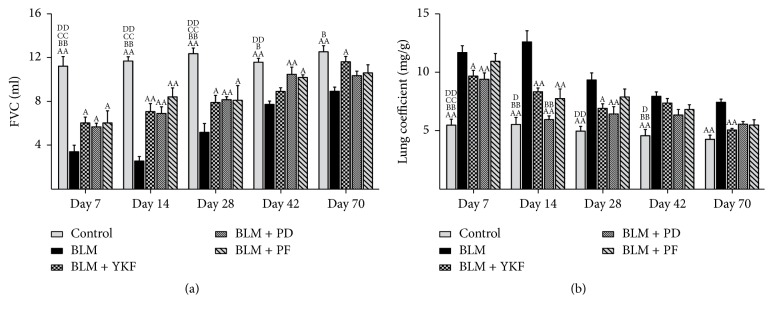
Changes of FVC and lung coefficient in bleomycin-induced pulmonary fibrosis rats treated with Yangqing Kangxian formula, prednisone, or pirfenidone. (a) Forced vital capacity (FVC). (b) Lung coefficient. BLM: bleomycin; YKF: Yangqing Kangxian formula; PD: prednisone; PF: pirfenidone. Values represented as mean ± SEM. ^AA^*P* < 0.01 and ^A^*P* < 0.05, versus BLM group. ^BB^*P* < 0.01 and ^B^*P* < 0.05, versus BLM + YKF group, ^CC^*P* < 0.01 and ^C^*P* < 0.05, versus BLM + PD group, and ^DD^*P* < 0.01 and ^D^*P* < 0.05, versus BLM + PF group.

As we know, progressive aggravation of pulmonary function is a feature of IPF. To elucidate the effects of YKF on BLM-induced pulmonary dysfunction, FVC was tested dynamically. As a result, FVC was significantly decreased in the BLM group rats. Compared with BLM rats, it was improved in YKF, prednisone, and pirfenidone treated groups throughout the experiment at different degrees. Moreover, FVC increased significantly in YKF treated rats on day 70 (*P* < 0.05). There was no significant difference among the three treatment groups (Figure 1(a)).

### 3.3. YKF Alleviated Pulmonary Injuries and Fibrosis in Rats

Lung coefficient is an index for evaluating lung edema. BLM exposure resulted in notable increase of lung coefficient from day 7 to day 70 (*P* < 0.01). Compared with BLM group, it was markedly decreased in YKF group (days 7, 14, 28, and 70, *P* < 0.05 or *P* < 0.01), prednisone group (days 7, 14, and 28, *P* < 0.01), and pirfenidone (day 14, *P* < 0.01). Compared with YKF group, lung coefficient of prednisone group is lower on day 14 (*P* < 0.01) (Figure 1(b)).

**Figure 2:**
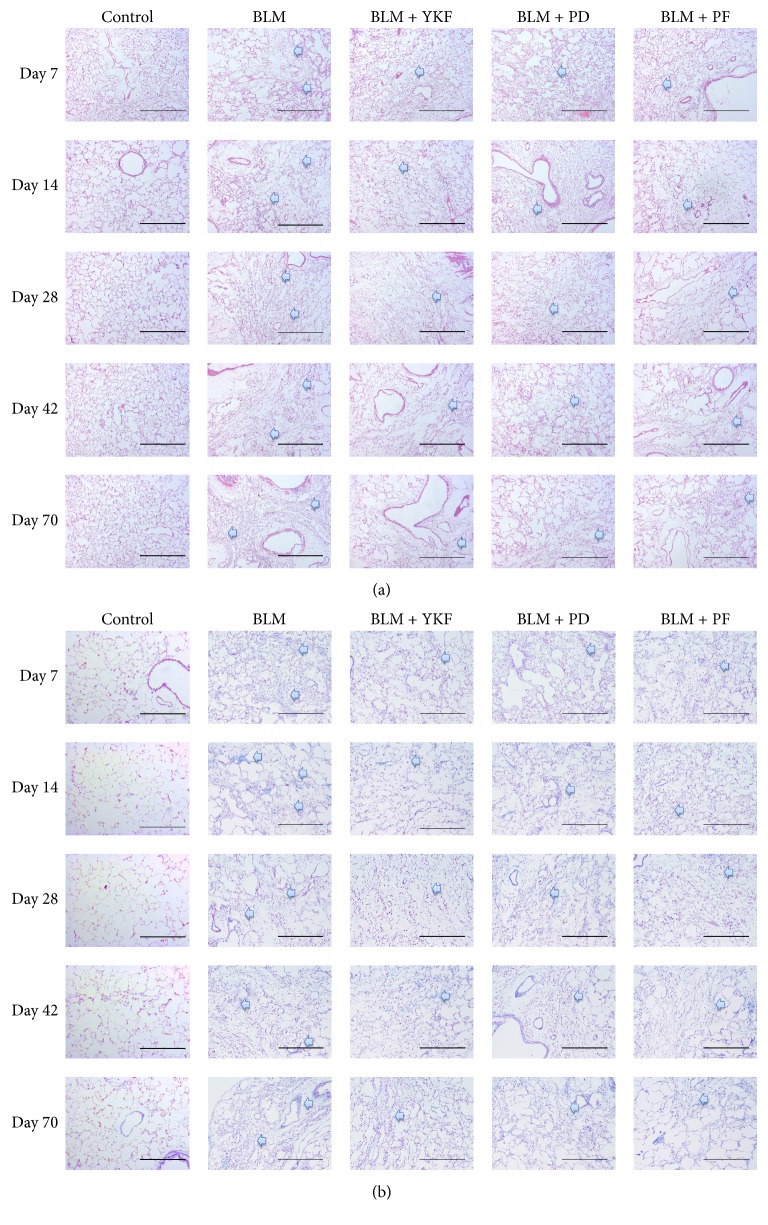
Representative histological images in bleomycin-induced pulmonary fibrosis rats treated with Yangqing Kangxian formula, prednisone, or pirfenidone. (a) Hematoxylin and eosin (H&E) staining, magnification 100x, scale bar: 200 *μ*m. (b) Masson trichrome staining, magnification 100x, scale bar: 200 *μ*m. BLM: bleomycin; YKF: Yangqing Kangxian formula; PD: prednisone; PF: pirfenidone. The blue arrows point to the lesion area.

**Figure 3:**
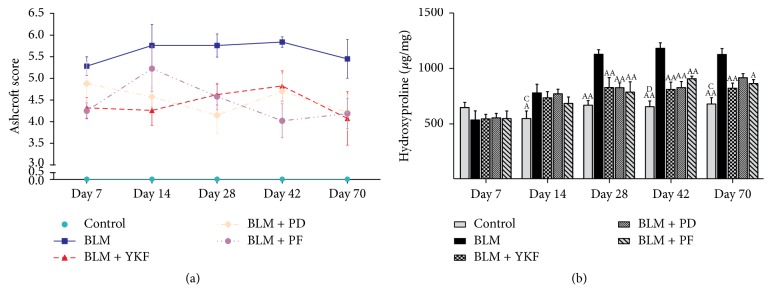
Changes of Ashcroft score and hydroxyproline in bleomycin-induced pulmonary fibrosis rats treated with Yangqing Kangxian formula, prednisone, or pirfenidone. (a) Ashcroft score. (b) Hydroxyproline (HYP). BLM: bleomycin; YKF: Yangqing Kangxian formula; PD: prednisone; PF: pirfenidone. Values represented as mean ± SEM. ^AA^*P* < 0.01 and ^A^*P* < 0.05, versus BLM group. ^BB^*P* < 0.01 and ^B^*P* < 0.05, versus BLM + YKF group, ^C^*P* < 0.05, versus BLM + PD group, and ^DD^*P* < 0.01 and ^D^*P* < 0.05, versus BLM + PF group.

To identify the degree of lung injury after treatment, sections of lung tissue were stained with H&E and Masson trichrome, and the severity of pulmonary fibrosis was assessed according to Ashcroft score. Normal structure with no pathologic changes was displayed in control rats under microscope. Extensive inflammatory infiltration, characterized by neutrophil and macrophage accumulation, and thickening of alveolar walls were observed obviously in parenchyma in IPF rats from day 7 to day 14 after bleomycin was challenged on day 0. Meanwhile, fibrosis region with marked disruption of the alveolar unit and accumulated deposition of collagen were also observed on day 7 and aggravated from day 14. On days 28, 42, and 70, consolidated areas of fibrosis accompanied with collagen accumulation were evident. The inflammation was marked and suppressed in rats treated with YKF, prednisone, or pirfenidone from 7 to 70 days after BLM was challenged, as well as the decrease in Ashcroft scores for fibrosis (Figures 2(a), 2(b), and 3(a)).

Pulmonary fibrosis is characterized by collagen accumulation. HYP is an index of collagen contents. Analyses of HYP contents were conducted to evaluate the effect of YKF. HYP was elevated in BLM challenged rats from day 28 up to day 70. Compared with BLM group, it was significantly decreased in YKF, prednisone, and pirfenidone group at different levels from day 28 up to day 42 (*P* < 0.01). Compared with BLM rats, HYP level reduced obviously in YKF and pirfenidone treated rats (*P* < 0.05) but not in prednisone treated rats (*P* > 0.05) on day 70. There was no significant difference among the three treatment groups at each time point (Figure 3(b)).

### 3.4. YKF Regulated Inflammatory Response

**Figure 4:**
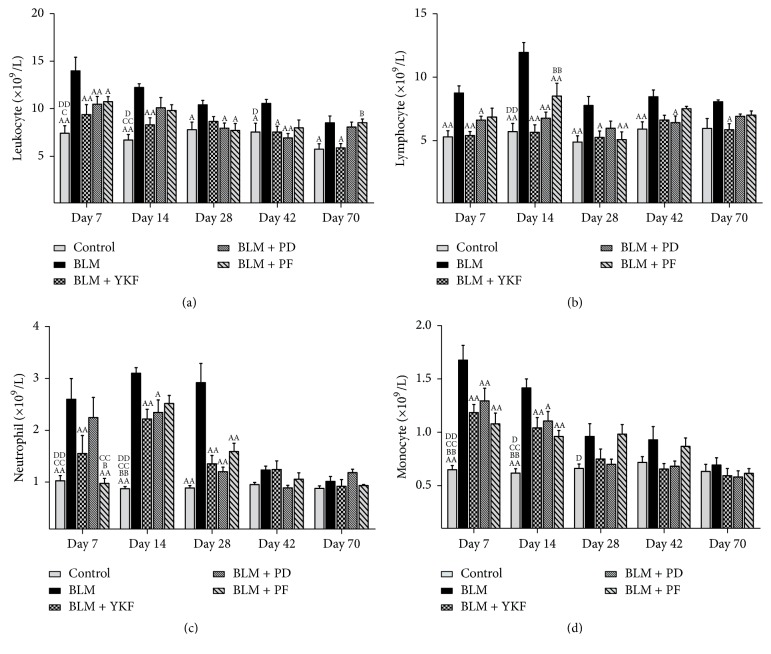
Changes of blood inflammatory cells population in bleomycin-induced pulmonary fibrosis rats treated with Yangqing Kangxian formula, prednisone, or pirfenidone. (a) Leukocyte. (b) Lymphocyte. (c) Neutrophil. (d) Monocyte. BLM: bleomycin; YKF: Yangqing Kangxian formula; PD: prednisone; PF: pirfenidone. Values represented as mean ± SEM. ^AA^*P* < 0.01 and ^A^*P* < 0.05, versus BLM group. ^BB^*P* < 0.01 and ^B^*P* < 0.05, versus BLM + YKF group, ^CC^*P* < 0.01 and ^C^*P* < 0.05, versus BLM + PD group, and ^DD^*P* < 0.01 and ^D^*P* < 0.05, versus BLM + PF group.

The numbers of blood inflammatory cells were analyzed by automated differential cell counter. As a result, BLM challenge resulted in significant increases in leukocyte populations from day 7 through day 70 (*P* < 0.05 or *P* < 0.01), while there were tendencies of suppression in YKF group (days 7, 14, 42, and 70, *P* < 0.05 or *P* < 0.01), prednisone group (days 7, 28, and 42, *P* < 0.05 or *P* < 0.01), and pirfenidone group (days 7 and 28, *P* < 0.05). Compared with YKF group, the leukocyte population of pirfenidone group is higher on day 70 (*P* < 0.05) (Figure 4(a)).

Compared with BLM group, lymphocyte level of YKF group decreased significantly on days 7, 14, 28, and 70 (*P* < 0.05 or *P* < 0.01); lymphocyte population decreased significantly in YKF group (days 7, 14, and 42, *P* < 0.05 or *P* < 0.01), prednisone group (days 7, 14, and 28, *P* < 0.05 or *P* < 0.01), and pirfenidone treated group (day 14, *P* < 0.01). Compared with YKF group, lymphocyte level is higher in pirfenidone group on day 14 (*P* < 0.01) (Figure 4(b)).

Neutrophils increased significantly in bleomycin group from day 7 to day 28 (*P* < 0.01). Compared with BLM group, the neutrophils level significantly decreased in YKF (on days 7, 14, and 28, *P* < 0.01), prednisone (on days 14 and 28, *P* < 0.05 or *P* < 0.01), and pirfenidone group (on days 7 and 28, *P* < 0.01). The neutrophil amount was greater in YKF group than pirfenidone group on day 7 (*P* < 0.05). Compared with prednisone group, the neutrophil level was lower in pirfenidone group on day 7 (Figure 4(c)).

Monocytes increased on days 7 and 14 in bleomycin challenged rats (*P* < 0.01) compared with BLM group; they were significantly decreased in YKF, prednisone, and pirfenidone groups on days 7 and 14 (*P* < 0.05 or *P* < 0.01) (Figure 4(d)). There was no significant difference among the three treatment groups (Figure 4(d)).

**Figure 5:**
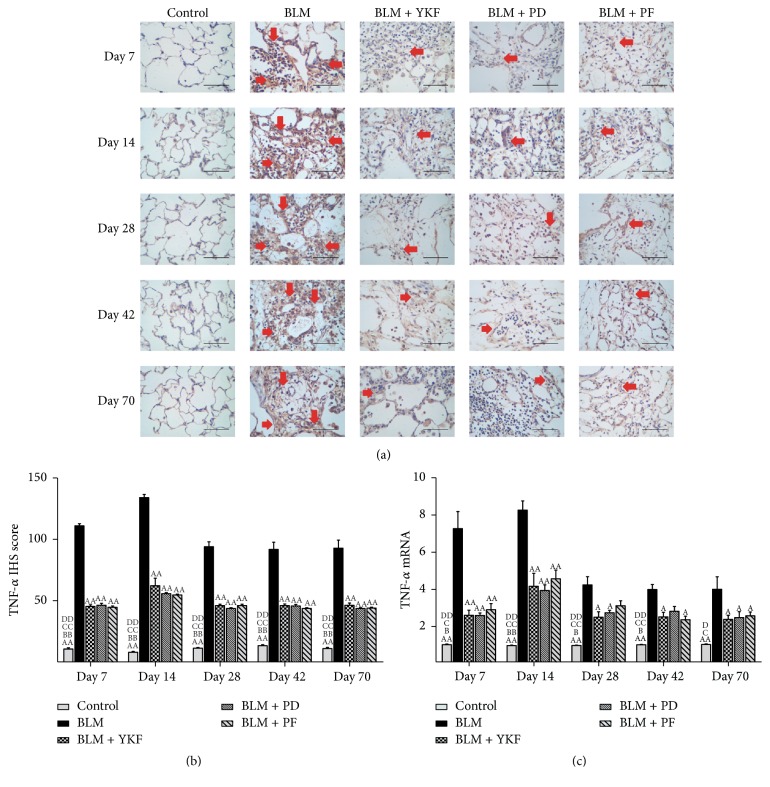
The expression of TNF-*α* protein and mRNA in bleomycin-induced pulmonary fibrosis rats treated with Yangqing Kangxian formula, prednisone, or pirfenidone. (a) Immunohistochemical stain of TNF-*α* protein, magnification 400x, scale bar: 50 *μ*m; (b) TNF-*α* protein; (c) TNF-*α* mRNA. BLM: bleomycin; YKF: Yangqing Kangxian formula; PD: prednisone; PF: pirfenidone. Values represented as mean ± SEM. ^AA^*P* < 0.01 and ^A^*P* < 0.05, versus BLM group. ^BB^*P* < 0.01 and ^B^*P* < 0.05, versus BLM + YKF group, ^CC^*P* < 0.01 and ^C^*P* < 0.05, versus BLM + PD group, and ^DD^*P* < 0.01 and ^D^*P* < 0.05, versus BLM + PF group. The red arrows point to the positive expression area.

**Figure 6:**
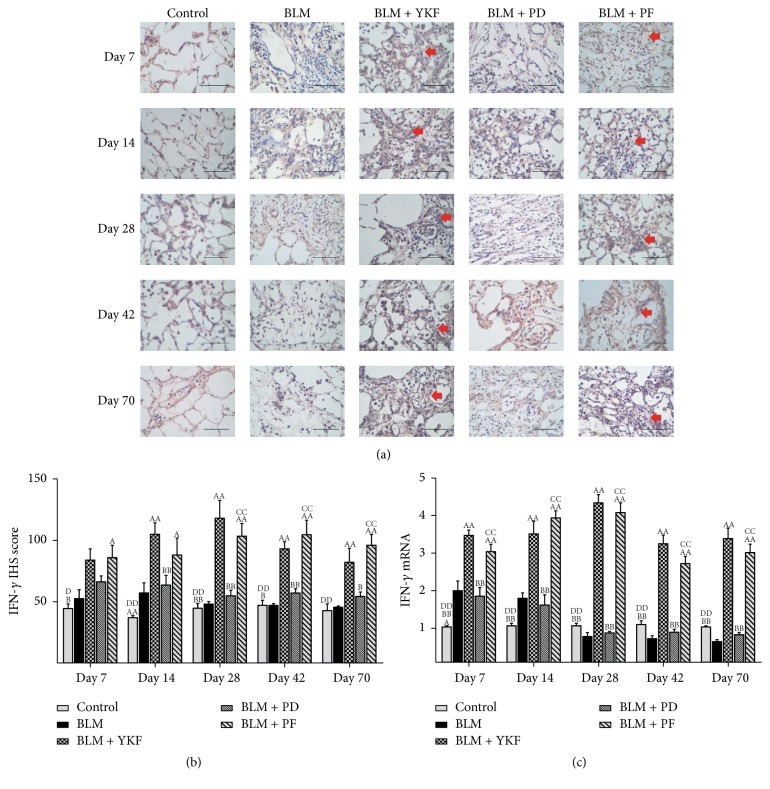
The expression of IFN-*γ* protein and mRNA in bleomycin-induced pulmonary fibrosis rats treated with Yangqing Kangxian formula, prednisone, or pirfenidone. (a) Immunohistochemical image of IFN-*γ*, magnification 400x, scale bar: 50 *μ*m; (b) IFN-*γ* protein; (c) IFN-*γ* mRNA. BLM: bleomycin; YKF: Yangqing Kangxian formula; PD: prednisone; PF: pirfenidone. Values represented as mean ± SEM. ^AA^*P* < 0.01 and ^A^*P* < 0.05, versus BLM group. ^BB^*P* < 0.01 and ^B^*P* < 0.05, versus BLM + YKF group, ^CC^*P* < 0.01 and ^C^*P* < 0.05, versus BLM + PD group, and ^DD^*P* < 0.01 and ^D^*P* < 0.05, versus BLM + PF group. The red arrows point to the positive expression area.

**Figure 7:**
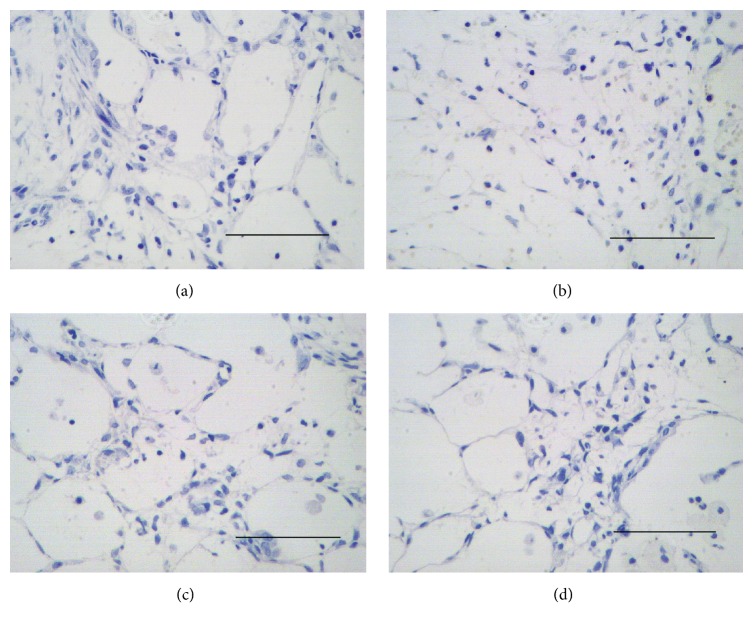
Negative control immunohistochemical image of TNF-*α* and IFN-*γ*, magnification 400x, scale bar: 50 *μ*m. ((a) and (b)) Negative control immunohistochemical image of TNF-*α*. ((c) and (d)) Negative control immunohistochemical image of IFN-*γ*.

**Figure 8:**
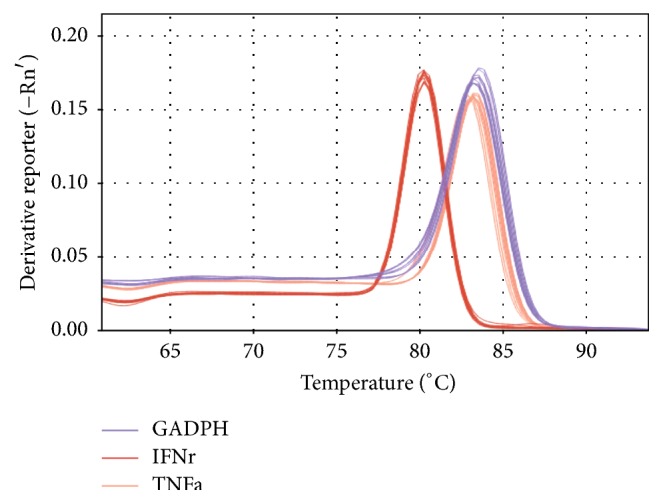
Melt curve plots of TNF-*α* and IFN-*γ*.

The inflammatory cytokine TNF-*α* and IFN-*γ* protein and mRNA in lung tissue were examined by immunohistochemical stain and RT-PCR. From day 7 to day 70, the expression of TNF-*α* protein and mRNA markedly increased in BLM challenged rats (*P* < 0.01) and significantly decreased in YKF, prednisone, and pirfenidone treated rats (Figures 5(a)~5(c)). There was no significant difference among the three treatment groups (Figures 5(a)–5(c), Figures 7(a)-7(b), and Figure 8). The expression of IFN-*γ* protein and mRNA increased from day 7 to day 14 in bleomycin challenged rats and decreased from day 28 to day 70. YKF and pirfenidone significantly increased the expression of IFN-*γ* from day 7 to day 70; however, prednisone did not work on regulating the expression of IFN-*γ* at each time point (*P* > 0.05). The increased expressions of IFN-*γ* mRNA induced by bleomycin were inhibited by the treatments of YKF and pirfenidone (*P* < 0.01) (Figures 6(a)–6(c), Figures 7(c)-7(d), and Figure 8).

**Figure 9:**
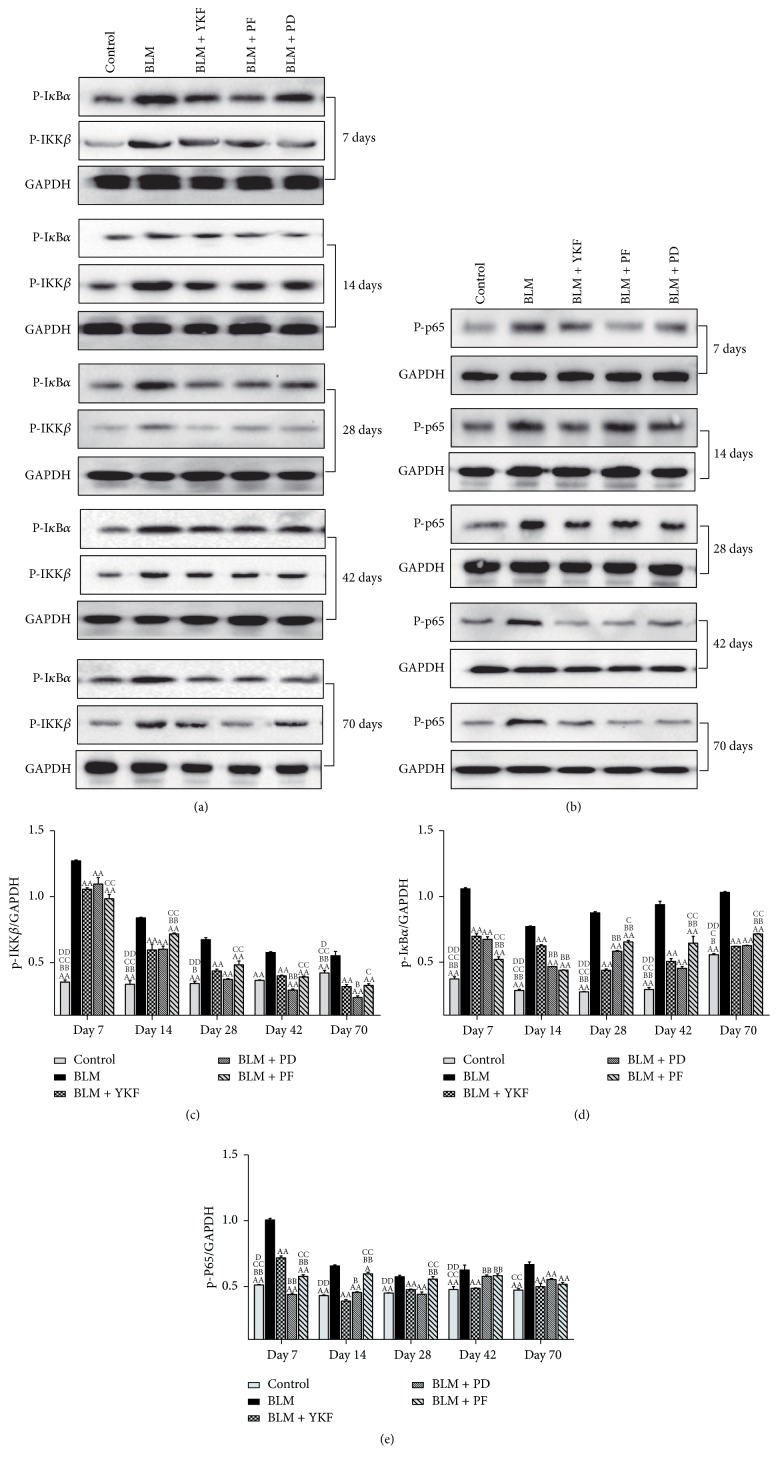
Phosphorylation protein levels of cytoplasm IKK*β* and I*κ*B*α* and nuclear NF-*κ*B in bleomycin-induced pulmonary fibrosis rats treated with Yangqing Kangxian formula, prednisone, or pirfenidone. (a) Phosphorylations of cytoplasm IKK*β* and I*κ*B*α* protein; (b) phosphorylations of nuclear NF-*κ*B p65 protein; (c) phosphorylations of cytoplasm IKK*β* protein; (d) phosphorylations of cytoplasm I*κ*B*α* protein; (e) phosphorylations of nuclear NF-*κ*B p65 protein. BLM: bleomycin; YKF: Yangqing Kangxian formula; PD: prednisone; PF: pirfenidone. *N* = 3. Values represented as mean ± SEM. ^AA^*P* < 0.01 and ^A^*P* < 0.05, versus BLM group. ^BB^*P* < 0.01 and ^B^*P* < 0.05, versus BLM + YKF group. ^CC^*P* < 0.01 and ^C^*P* < 0.05, versus BLM + PD group; ^DD^*P* < 0.01 and ^D^*P* < 0.05, versus BLM + PF group.

NF-*κ*B signal pathway is an important pathway and has been proved to be excessively activated in the inflammation response. NF-*κ*B signal pathway is activated in lung tissue with bleomycin challenge. To further clarify the role of YKF on BLM-induced pulmonary injury, activated proteins related to NF-*κ*B signal pathway were detected by Western blotting. As the results shown in Figures 9(a) and 9(c), there was significant elevation of the expression of p-IKK*β* protein after bleomycin challenge from day 7 to day 70 (*P* < 0.01), while it was markedly suppressed in YKF, prednisone and pirfenidone treated rats (*P* < 0.01). Compared with pirfenidone and prednisone group, the expression of p-IKK*β* decreased in YKF group at different levels throughout the experiment (days 14, 42, and 70: *P* < 0.05 or *P* < 0.01). As shown in Figures 9(a) and 9(d), the expression of p-I*κ*B*α* protein was increased significantly in bleomycin challenged rats from day 7 to day 70 (*P* < 0.01), and it was markedly suppressed in YKF, prednisone, and pirfenidone groups (*P* < 0.01). Compared with pirfenidone and prednisone group, p-I*κ*B*α* decreased in YKF group at different levels throughout the experiment (days 7, 14, 28, 42, and 70: *P* < 0.01). Results in Figures 9(b) and 9(e) showed that the expression of p-P65 protein was significantly increased in bleomycin challenged rats from day 7 to day 70 (*P* < 0.01) and was markedly suppressed in YKF, prednisone, and pirfenidone treated rats (*P* < 0.01). Compared with pirfenidone and prednisone group, p-P65 decreased in YKF group at different levels throughout the experiment (days 7, 14, 28, and 42: *P* < 0.05 or *P* < 0.01).

## 4. Discussion

IPF is a chronic intractable disease. There are no effective therapies for the progressive disorders of pulmonary mechanics and respiratory function, which are induced by lung tissue injury and repair. Many patients are still suffering from short survival time [29]. According to the TCM theory, IPF belongs to the category of Feiwei Disease. In the earlier stage, the main syndrome is characterized by the deficiency of qi and yin and the stasis of heat and phlegm [30–32]. Based on TCM theory and data from previous literature studies, we designed the Yangqing Kangxian formula, which can tonify qi and yin, promote blood circulation, remove blood stasis, clear heat, and dissipate phlegm. In clinical treatment of IPF patients, we found that YKF had beneficial effects on alleviating the clinical symptoms of IPF patients. So it is important to examine and clarify the effect of this candidate TCM formula.

Bleomycin-induced pulmonary fibrosis model is used in previous research. Initially, BLM was found to be effective in squamous cell carcinoma and skin tumors, so it was used in the treatment of tumor. With increasing clinical use, the side effect was gradually recognized by the doctors [33]. Because of pulmonary fibrosis and the serious toxicity of this drug, BLM was widely used in the establishment of animal pulmonary fibrosis model. In response to bleomycin-induced pulmonary injury, a large increase in inflammatory cells infiltration, thickened alveolar walls, excessive collagen deposition, accumulated fibroblast proliferation, and severe distortion of alveolar structure have been seen in the progression of the disease [34, 35]. As previous studies and our preliminary experiment results showed, chronic fibrosis induced by BLM can exist for a long time [25, 34]. Thus, BLM-induced pulmonary fibrosis model is also applied to long-term observation.

Here, we found that marked inflammatory cells infiltration, collagen deposition, and HYP level elevation were observed in our research, and the decline of respiratory function was induced by these progressive pathological changes. The administration of YKF has many beneficial effects, such as improving FVC, alleviating pulmonary injuries, and reducing fibrosis degree, and it still has long-term effect after withdrawal. The extent of amelioration by YKF is similar to that afforded by prednisone (a drug widely used to treat IPF at one time) or pirfenidone (a drug conditionally recommended in clinical practice guideline). In the present study, the increased inflammatory cells populations in blood were suppressed by the treatment of YKF. The results above suggest that YKF might contribute to regulating inflammatory response in the course of IPF.

Pulmonary fibrosis could be considered as the final outcome of inflammatory process in the lung. The inflammatory response following injury is crucial to the process of IPF. The response includes migration and activation of inflammatory cells and the release of certain cytokines. Fibroblasts multiplication, migration, and collagen production were stimulated by the cytokines. TNF-*α* and IFN-*γ* have been proved to be associated with the course of IPF [1, 6, 9, 10, 36–40]. Our results have shown that TNF-*α* was decreased in YKF treated rats lungs, and IFN-*γ* was increased. This suggested that the release of some inflammatory cytokines could be regulated by YKF.

For further underlying the mechanisms, we focused on NF-*κ*B signal pathway related molecules. NF-*κ*B signal pathway is an important pathway and has been proved to be excessively activated in the inflammation response. In previous studies, the activation of NF-*κ*B signaling was increased in lung tissue with bleomycin challenge [39, 41]. In the canonical pathway, cells are stimulated by factors such as TNF-*α*, followed by the activation of IKK*β*, I*κ*B*α*, and NF-*κ*B; NF-*κ*B nuclear translocation and phosphorylation regulate proinflammatory gene expression and increase the production of various inflammatory cytokines [42, 43]. In this study, we focused on the phosphorylations of cytoplasm IKK*β* and I*κ*B*α* and nuclear NF-*κ*B [8] and found that the levels of p-IKK*β*, p-I*κ*B*α*, and p-NF-*κ*B were upregulated in bleomycin rats at each time point and were downregulated by the administration of YKF. The results above suggest that anti-inflammatory response and alleviating fibrosis effects of YKF contribute to its protective effect against pulmonary fibrosis.

## 5. Conclusions

Taken together, YKF has beneficial protective and long-term effects on pulmonary fibrosis by anti-inflammatory response and alleviating fibrosis. Thus, administration of YKF might be an effective therapy to pulmonary fibrosis.

## Figures and Tables

**Table 1:** The main compositions and chemical compounds of Yangqing Kangxian formula.

Main composition	Latin name	Main chemical compounds	Amount (g)
Mai Men Dong	*Ophiopogon japonicas*	Ophiopogonin A, methylophiopogonanone A	15
Nan Sha Shen	*Adenophorae Ae *Radix	Mandanol, beta-sitosterol, ethyl oleate (NF)	12
Xi Yang Shen	*Panax quinquefolius *Radix	Polyacetylene PQ-2, beta-sitosterol, papaverine	6
Gua Lou	*Trichosanthes kirilowii *Maxim	Diosmetin, spinasterol, hydroxygenkwanin	15
Zhe Bei Mu	*Fritillariae thunbergii *Bulbus	Beta-sitosterol, pelargonidin, zhebeiresinol	9
Chi Shao	*Radix Paeoniae *Rubra	Baicalein, evofolin B, paeoniflorigenone	12

**Table 2:** Treatment protocol for the experiment.

	Treatment phase				Treatment-free phase			
Group	(Day 1 to day 42)				(Day 43 to day 70)			
	NS	NS	PD	PF	NS	NS	PD	PF
Control	+	+	−	−	−	−	−	−
BLM	+	+	−	−	−	−	−	−
BLM + YKF	−	−	−	−	−	−	−	−
BLM + PD	−	−	+	−	−	−	−	−
BLM + PF	−	−	−	+	−	−	−	−

*Note*. +: treated with this medicine; −: not treated with this medicine; BLM: bleomycin-induced IPF rats; BLM + YKF: bleomycin-induced IPF rats treated with Yangqing Kangxian formula; BLM + PD: bleomycin-induced IPF rats treated with prednisone; BLM + PF: bleomycin-induced IPF rats treated with pirfenidone.

**Table 3:** The primer sequences of mRNAs.

Gene		Oligonucleotide primers (5′-3′)	Product size (bp)	Annealing (°C)	Cycle
TNF-*α*	F	CGTCAGCCGATTTGCCATTT	88	60	40
	R	TCCCTCAGGGGTGTCCTTAG		60	40

IFN-*γ*	F	GAGGAACTGGCAAAAGGACG	132	60	40
	R	AGGTGCGATTCGATGACACT		60	40

GADPH	F	AAGGTCGGTGTGAACGGATT	70	60	40
	R	CTTTGTCACAAGAGAAGGCAGC		60	40

*Note*. F: forward; R: reverse.

**Table 4:** Mortalities of the rats in each group.

	*N*	Number of deaths						Mortality (%)
		Day 0	Days 1 to 7	Days 8 to 14	Days 15 to 21	Days 21 to 28	Days 29 to 70	
Group	40	0	0	0	0	0	0	0
BLM	40	2	6	4	0	0	0	30
BLM + YKF	40	1	4	4	0	1	0	25
BLM + PD	40	1	5	5	0	0	0	27.5
BLM + PF	40	0	4	5	1	0	0	25

*Note*. BLM: bleomycin-induced IPF rats; BLM + YKF: bleomycin-induced IPF rats treated with Yangqing Kangxian formula; BLM + PD: bleomycin-induced IPF rats treated with prednisone; BLM + PF: bleomycin-induced IPF rats treated with pirfenidone.
